# An Integrated Study to Analyze Soil Microbial Community Structure and Metabolic Potential in Two Forest Types

**DOI:** 10.1371/journal.pone.0093773

**Published:** 2014-04-17

**Authors:** Yuguang Zhang, Jing Cong, Hui Lu, Caiyun Yang, Yunfeng Yang, Jizhong Zhou, Diqiang Li

**Affiliations:** 1 Institute of Forestry Ecology, Environment and Protection, and the Key Laboratory of Forest Ecology and Environment of State Forestry Administration, Chinese Academy of Forestry, Beijing, China; 2 School of Mineral Processing and Bioengineering, Central South University, Changsha, China; 3 Institute for Environmental Genomics and Department of Microbiology and Plant Biology, University of Oklahoma, Norman, Oklahoma, United States of America; 4 State Key Joint Laboratory of Environment Simulation and Pollution Control, School of Environment, Tsinghua University, Beijing, China; 5 Earth Sciences Division, Lawrence Berkeley National Laboratory, Berkeley, California, United States of America; U. S. Salinity Lab, United States of America

## Abstract

Soil microbial metabolic potential and ecosystem function have received little attention owing to difficulties in methodology. In this study, we selected natural mature forest and natural secondary forest and analyzed the soil microbial community and metabolic potential combing the high-throughput sequencing and GeoChip technologies. Phylogenetic analysis based on 16S rRNA sequencing showed that one known archaeal phylum and 15 known bacterial phyla as well as unclassified phylotypes were presented in these forest soils, and *Acidobacteria*, *Protecobacteria*, and *Actinobacteria* were three of most abundant phyla. The detected microbial functional gene groups were related to different biogeochemical processes, including carbon degradation, carbon fixation, methane metabolism, nitrogen cycling, phosphorus utilization, sulfur cycling, etc. The Shannon index for detected functional gene probes was significantly higher (*P*<0.05) at natural secondary forest site. The regression analysis showed that a strong positive (*P*<0.05) correlation was existed between the soil microbial functional gene diversity and phylogenetic diversity. Mantel test showed that soil oxidizable organic carbon, soil total nitrogen and cellulose, glucanase, and amylase activities were significantly linked (*P*<0.05) to the relative abundance of corresponded functional gene groups. Variance partitioning analysis showed that a total of 81.58% of the variation in community structure was explained by soil chemical factors, soil temperature, and plant diversity. Therefore, the positive link of soil microbial structure and composition to functional activity related to ecosystem functioning was existed, and the natural secondary forest soil may occur the high microbial metabolic potential. Although the results can't directly reflect the actual microbial populations and functional activities, this study provides insight into the potential activity of the microbial community and associated feedback responses of the terrestrial ecosystem to environmental changes.

## Introduction

Microorganisms are the most abundant organisms on earth and play key roles in natural ecosystem, including the biogeochemical cycling of carbon, nitrogen, sulfur, phosphorus, and metals, and biodegradation or stabilization of environmental contaminants [1]–[3]. Because of their important roles, changes in soil microbial community may directly affect soil ecosystem function, particularly carbon and nitrogen cycling [4]. A deeply analysis of microbial community structure and their roles in ecological processes would improve our understanding of the biogeochemical elemental cycles affected by microbial communities in natural or man-made environments [5]. However, our understanding of soil microbial communities in terms of structure, composition, and functional activity are still limited, especially for soil microbial metabolic activity and ecosystem function have received little attention to date.

Numerous studies have investigated the structure and diversity of microbial communities and their relationships with the surrounding environments [4]–[8]. Previous studies have shown inconsistent or contradictory results, which might be caused by 1) high microbial community complexity and plasticity in natural environments, 2) different methods used to assess microbial diversity, 3) frequency and magnitude of environmental disturbance, and 4) limited knowledge on the temporal and spatial scales of microbial community in ecosystems [4]–[7], [9]. To date, most studies have only described microbial community complexity at the phylogenetic level using 16S rRNA or single functional gene analysis [8]. Further studies of the metabolic potential and physiological traits of microorganisms are necessary to detect specific metabolic processes and functions [4]. Functional gene microarrays, with quantitative and high resolution capabilities, may be a useful tool for this purpose [10], [11].

With the increasing efforts made towards implementing sustainable forest management and conservation strategies since last century [12], natural secondary forest, which form naturally after disturbance of the primary forest by human activities or by extreme natural events, are evident in temperate regions around the world [13]. Shennongjia Mountain is located in the northwestern region of Hubei Province, Central China, and this region is considered to have one of the highest levels of biodiversity in China owing to its unique geographic location and complex terrain [14]. In this study, we selected natural mature forest and a natural secondary forest, which formed in the 1970s following the fall of the primary deciduous broadleaf forest on Shennongjia Mountain and analyzed the soil microbial community and metabolic potential using both GeoChip and high-throughput 16S rRNA sequencing approaches. The aims of this study were to determine (i) the composition and structure of soil microbial community in two deciduous broadleaf forest types, (ii) the soil microbial functional gene diversity and the metabolic potential involved in carbon and nitrogen cycling, and (iii) the major environmental factors governing the soil microbial community structure.

## Materials and Methods

### Site and sampling

The study site was located on Shennongjia Mountain, Hubei Province, China, where deciduous broadleaf forest is a typical and important forest type. Two forest types were selected in this study: natural mature forest (MAT), and natural secondary forest (SEC), which was formed in the 1970s following the felling of the primary deciduous broadleaf forest. The two study sites had similar geography in terms of slope (30°), aspect (NE30°), and elevation (1715–1813 m), and were within 10 km of each other. The soil type was mountain yellow brown soil [15]. The plant survey and soil collected were permitted by the administrative bureau of Shennongjia National Nature Reserve. The detail location for the study site is N 31°29′, E110°21′ for MAT and N 31°25′, E11°20′ for SEC, respectively. At each study site, four plots (20 m×20 m) were selected with about 20 meters between adjacent plots. Ten to fifteen soil cores, at a depth of 0–10 cm, were taken from each plot and combined to obtain about 400 g of soil. Samples were sieved with 2 mm mesh to remove roots and stones, then mixed thoroughly. Soil samples were preserved at −80°C until DNA extraction.

### Plant diversity, soil geochemical properties and microbial biomass analyses

Plant diversity was surveyed at each plot, including the plant species, number, height and canopy of each tree or shrub, and diameter at breast (1.3 m) height of trees (DBH>5 cm) and shrubs (DBH>1 cm). Average soil temperature at each plot was measured by placing the Long-Stem Thermomter (SPECTRUM, US) at a depth of 10 cm in relatively open patches. Soil moisture, soil pH, total soil organic carbon and nitrogen, available phosphorous, available water-dissolved organic carbon, and oxidizable organic carbon were measured as previously described [16]. Microbial biomass carbon was determined by the chloroform fumigation-extraction method [17].

### DNA extraction, purification, and quantification

Soil microbial genomic DNA was extracted directly from each soil sample (5 g) using a protocol that included liquid nitrogen grinding, freezing and thawing, and treatment with sodium dodecyl sulfate for cell lysis, as previously described [18]. The freshly extracted DNA was purified twice using 0.5% low melting point agarose gel. Final DNA concentrations were quantified by the PicoGreen method [19] using a FLUOstar Optima microplate reader (BMG Labtech, Jena, Germany).

### DNA amplification, sequencing, and data analysis

The V4 hypervariable region of the 16S rRNA gene was amplified in each soil sample using PCR primers 515F (5′-GTGCCAGCMGCCGCGGTAA-3′) and 806R (5′-GGACTACH VGGGTWTCTAAT-3′), using a sample tagging approach [20], [21]. The amplification mix contained 10 units of AccuPrime High Fidelity *Taq* polymerase (Invitrogen, Grand Island, NY), 1× AccuPrime PCR buffer II (Invitrogen), 200 µM dNTPs (Amersham, Piscataway, NJ), and 10 µM of each primer in a volume of 25 µl. Genomic DNA (10 ng) was added to the PCR mix. Each sample was amplified under the following conditions: 94°C for 1 min, 30 cycles of 94°C for 20 s, 53°C for 25 s, and 68°C for 45 s, then 10 min at 68°C. The purified PCR amplicons were analyzed using a Miseq Benchtop Sequencer for 2×150 bp paired-end sequencing (Illumina, San Diego, CA).

To minimize the effect of random sequencing errors, only the first 250 bp after the proximal PCR primer of each sequence was analyzed. Sequence that did not perfectly match the PCR primer, had non-assigned tags, or had reads <250 bp was removed. All sequences were aligned using the Ribosomal Database Project Infernal Aligner [22], and the complete linkage clustering method was used to define operational taxonomic units (OTUs) using 97% identity as a cutoff [21]. The number of detected OTUs and sequences at different levels of classification were counted. Details of amplicon preparations, sequencing, and data analysis (e.g. classification, OTU identification) were described in previous reports [20], [21]. To standardize samples, a sub-sample of 20,000 sequences per soil sample was used to compare relative difference among samples. Singletons were removed for downstream analyses. All the 16S rRNA sequences were deposited in GenBank database and the accession number is SRP035449.

### GeoChip hybridization and data analysis

GeoChip 4.0 was used for DNA hybridization. This microarray contains >83,000 oligonucleotide probes targeting >150,000 genes in 410 gene categories involved in biogeochemical cycling of carbon, nitrogen, phosphorus, and sulfur, and bioremediation of metal and organic contaminants [24]. To produce consistent hybridizations from all samples, the amplification was used to generate approximately 3.0 µg of DNA from 50 ng of purified DNA using a TempliPhi Kit (GE Healthcare, Piscataway, NJ) following the manufacturer's instructions. Amplified DNA was labeled with Cy5 fluorescent dye (GE Healthcare) using a random priming method [23]. All hybridizations were carried out at 45°C for 10 h with 50% formamide using a TECAN HS4800 (TECAN, US) microarray hybridization station, and arrays were scanned using a ScanArray 5000 analysis system (Perkin-Elmer, Wellesley, MA).

Signal intensities of each spot were measured using ImaGene 6.0 (Biodiscovery, El Segundo, CA), and only the spots automatically scored as positive in the raw data output were used for further data analysis [25]. Spots with a signal-to-noise ratio (SNR = (signal intensity - background intensity)/standard deviation of the background) greater than 2.0 were used for further analysis [23]. The GeoChip data were further analyzed using the following steps: (i) genes detected in only two out of four samples from the same forest type were removed; (ii) the signal intensity of each spot was normalized by dividing by the mean value of eight samples of total signal intensity; and (iii) the data were transformed to the natural logarithmic form.

### Statistical analyses

Relative abundance of the 16S rRNA gene was based on the proportional frequencies of those DNA sequences that could be classified at different OTUs. Diversity of the microbial community based on sequencing analysis and diversity of functional gene from GeoChip analysis were calculated using Simpson's reciprocal index (1/D) and the Shannon index (H′) using online software (http://ieg.ou.edu/). Detrended correspondence analysis (DCA) was used to determine the differences in overall microbial community structure and functional gene diversity between the two forest types. To examine the differences in soil microbial community structure and functional diversity between the two study sites, we used the Bray-Curtis similarity index to calculate distance matrices from OTUs and GeoChip hybridization data with the multi-response permutation procedure (MRPP) [26] and Adonis [27]. Mantel tests, canonical correspondence analysis (CCA), and variation partitioning analysis were used to evaluate the linkages between microbial community structure, metabolic potential related to carbon and nitrogen cycles, and environmental factors. The variance inflation factor was used for step-wise removal of redundant variables in CCA modeling [23]. All the analyses were performed by functions in the Vegan package (v. 1.15-1) in R (v. 2.9.1) (http://www.r-project.org/).

## Results

### Plant diversity and soil geochemical properties

Plant diversity and soil properties in the MAT and SEC forests were analyzed (Table 1). The number of plant species (including trees and shrubs) was 63 and 91 in MAT and SEC, respectively. The dominant trees were *Fagus engleriana*, *Quercus aliena var. acuteserrata*, and *Carpinus viminea* at the MAT forest site, and *Toxicodendron verniciflua*, *Tilia paucicostata*, *Juglans cathayensis*, and *Q. aliena var. acuteserrata* at the SEC site, respectively. The Shannon index of plant was significantly lower for MAT (1.18) than for SEC (2.12).

**Table 1:** Plant diversity and soil geochemical properties in two forest types.

Forest type	MAT	SEC	Forest type	MAT	SEC
non index of tree and shrubs**	1.18±0.27	2.12±0.17	Soil organic carbon (g/kg)**	36.40±3.41	86.07±9.70
Species number of tree*	6.00±0.41	12.50±2.10	Dissolved organic carbon (g/kg)	0.17±0.01	0.26±0.08
Height of tree**	14.14±0.54	8.73±0.34	Liable organic carbon (g/kg)**	2.00±0.48	12.37±1.58
Diameter at breast height of tree**	19.85±1.02	12.06±0.75	Microbial biomass carbon (g/kg)**	0.81±0.05	1.31±0.07
Soil Moisture (%) **	50.12±1.23	27.32±2.45	Total nitrogen (g/kg)**	2.33±0.45	5.83±0.45
Temperature at 10 cm depth (°C)	15.92±0.15	15.32±0.09	Available nitrogen (g/kg)**	0.22±0.02	0.41±0.03
Soil pH**	4.81±0.05	5.91±0.28	Available phosphorus (g/kg) *	0.004±0.00	0.015±0.00

Data present the mean value and standard error.

*, *P*<0.05,

**, *P*<0.01.

Soil organic carbon, oxidizable organic carbon, and microbial biomass carbon were significantly higher (*P*<0.01) in the SEC samples than in the MAT samples. Soil geochemical properties, such as total nitrogen, available nitrogen, available phosphorus, pH, and soil moisture, were also significantly different (*P*<0.01) between the two forest types (Table 1).

### Soil microbial community composition and structure

To determine the overall composition of the soil microbial community in the two forest types, soil microbial communities were analyzed by 16S rRNA high throughput sequencing. After preprocessing of all reads, 65,666 and 65,584 high-quality sequences were obtained from the MAT and SEC sites, respectively (Table S1). The number of sequences ranged from 15,521 to 16,919 in MAT samples and from 16,002 to 16,970 in SEC samples. 10,160 OTUs were detected using 97% identity as a cutoff. Phylogenetic analysis showed that one known archaeal phylum and 15 known bacterial phyla as well as unclassified phylotypes were presented in these forest types (Table 2), and that all of the phylotypes were detected in both MAT and SEC, except for BRC1, which was only found in SEC. At the phylum level, the majority of OTUs were derived from *Proteobacteria* (4,048, 39.8%), followed by *Acidobacteria* (1,787, 17.6%), *Actinobacteria* (1,019, 10.0%), and *Verrucomicrobia* (896, 8.82%) (Table 2).

**Table 2:** Numbers of soil microbial OTUs and composition in two forest types.

Domain and phylum	Total^a^ (%)	Average^b^		P (unpaired t test)
		MAT	SEC	
***Archaea***				
*Crenarchaeota*	19 (0.19)	4.50±0.96	10.00±0.82	0.005
***Bacteria***				
*Acidobacteria*	1787 (17.59)	777.75±22.21	685.50±14.06	0.017
*Actinobacteria*	1019 (10.03)	362.25±19.69	443.75±17.09	0.021
*Armatimonadetes*	22 (0.22)	5.25±0.63	8.50±1.19	0.065
*Bacteroidetes*	621 (6.11)	91.50±5.87	379.50±10.81	0.000
BRC1	3 (0.03)	0.00	2.25±0.25	0.000
*Chlamydiae*	17 (0.17)	8.00±1.47	1.50±0.28	0.019
*Chloroflexi*	109 (1.07)	26.25±2.32	42.25±3.71	0.014
*Cyanobacteria*	3 (0.03)	1.00±0.00	2.50±0.29	0.014
*Firmicutes*	129 (1.27)	45.50±4.17	60.75±5.07	0.061
*Gemmatimonadetes*	97 (0.95)	23.00±1.08	53.50±5.74	0.011
*Nitrospirae*	7 (0.07)	1.00±0.41	4.50±0.29	0.001
*Planctomycetes*	657 (6.47)	288.50±9.91	173.75±9.29	0.000
*Proteobacteria*	4048 (39.84)	1409.00±31.39	1826.25±52.01	0.001
*Verrucomicrobia*	896 (8.82)	459.00±41.12	349.75±3.07	0.076
WS3	12 (0.12)	0.50±0.28	6.50±0.87	0.004
Unclassified	714 (7.03)	212.25±5.96	326.75±11.06	0.000
**Total**	**10160**	**3623.00±77.66**	**4469.75±53.03**	**0.000**

a Data represent total numbers of OTUs detected by sequencing across all 8 samples.

b Data represent the mean value and standard error of OTUs detected using 4 samples in different forest types.

**Figure 1:**
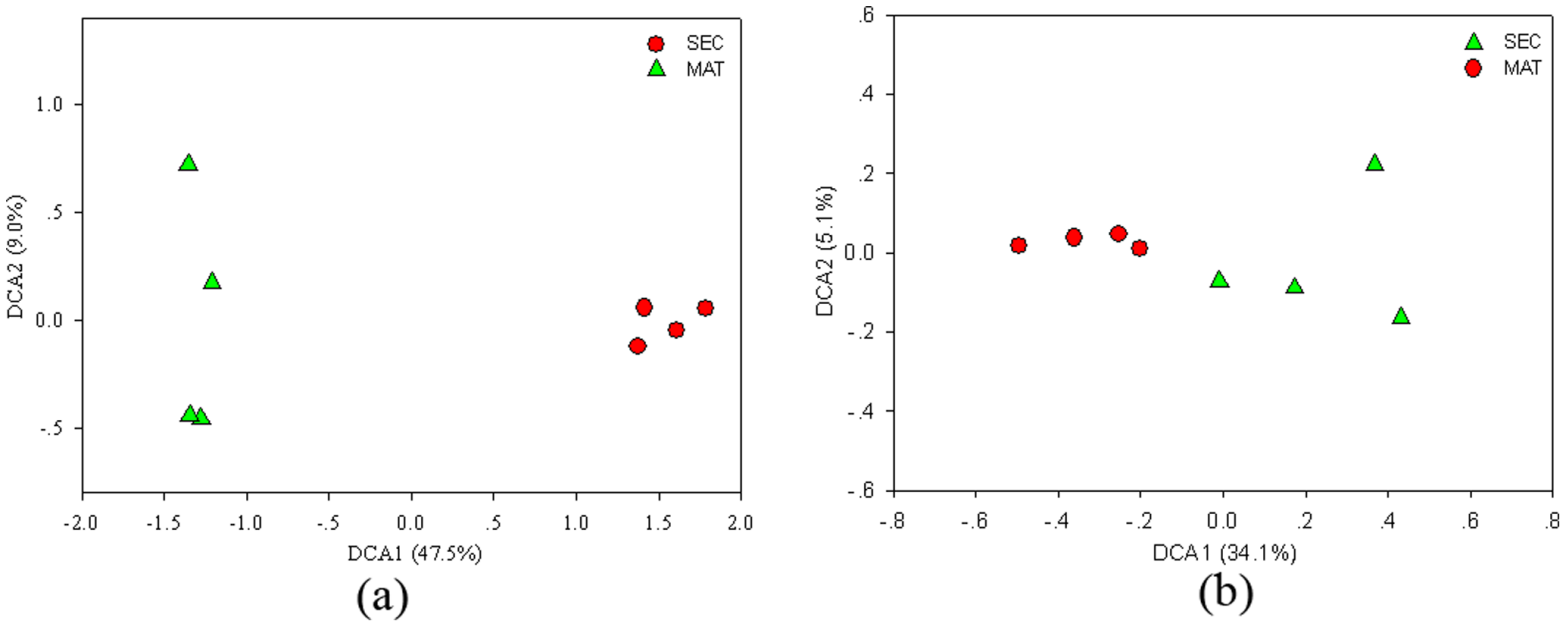
Detrended correspondence analysis (DCA) of soil microbial community structure. The DCA was analyzed based on the relative abundances of OTUs (A) and functional genes based on GeoChip 4.0 (B).

The Shannon index of 16S rRNA sequences was 8.05 and 8.28 for the MAT and SEC sites, respectively (Table S1). The number of OTUs and the Simpson index were significantly (*P*<0.05) different between MAT and SEC samples (Table S1). DCA was performed using the relative abundance values of sequencing data, resulting in two distinct clusters (Figure 1A). The results of nonparametric multivariate statistical tests, Adonis and MRPP, showed significant differences (R^2^ = 0.688, *P* = 0.001, and δ = 0.387, *P* = 0.026, respectively) based on the abundance of all OTUs detected in MAT and SEC samples (Table S2). Significant (*P*<0.05) differences were also observed at the phylum level for *Acidobacteria, Actinobacteria, Armatimonadetes, Bacteroidetes, Proteobacteria, Planctomycetes*, and BRC1, except for WS3 (Table 2). The differences in microbial diversity at the phylum level in the two forest types varied. Ten known microbial phyla and unclassified taxa were significantly (*P*<0.05) higher in SEC samples, including the dominant phyla *Proteobacteria* and *Actinobacteria*. However, *Acidobacteria*, *Chlamydiae*, and *Planctomycetes* were significantly (*P*<0.05) lower in SEC soils. These results indicated that the overall soil microbial taxonomic composition and structure were significantly different between the SEC and MAT samples.

### Soil microbial functional gene diversity

**Figure 2:**
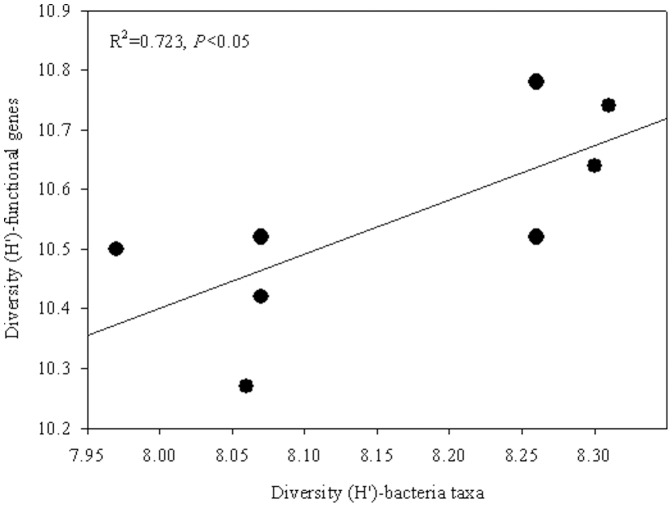
Relationship between soil microbial phylogenetic diversity index and functional gene diversity index.

A total of 38,925 and 50,068 gene probes were detected using GeoChip 4.0 in the MAT and SEC samples, respectively (Table S3). The detected gene groups were related to different biogeochemical processes, such as carbon degradation, carbon fixation, methane metabolism, nitrogen cycling, phosphorus utilization, stress responses, sulfur cycling, contaminant remediation, metal remediation, and energy processing (Table S4). The average number of gene probes detected was 43,427.3 for SEC samples and 33,982.8 for MAT samples. The Shannon index for detected functional microbial gene probes were significantly higher (*P*<0.05) at SEC sites (10.67) than at MAT sites (10.43) (Table S3). DCA showed that MAT samples were well separated from SEC samples (Figure 1B). The regression analysis showed that a strong positive (*P*<0.05) correlation was existed between the soil microbial functional gene diversity and phylogenetic diversity (Fig. 2).

### Microbial functional genes and metabolic potential related to carbon cycling

To gain insight into the soil microbial metabolic potential related to carbon cycle, GeoChip 4.0 data for genes related to carbon cycling was analyzed further. Among the 5,792 gene probes related to carbon degradation, 4,276 (74%) were common between the two forest types, while 59 (1%) were presented only in MAT samples and 1,457 (25%) were presented only in SEC samples. The relative signal intensities of the different gene categories related to degradation of relatively active carbon (e.g. starch, hemicelluloses, pectin, and cellulose) and recalcitrant carbon (e.g. chitin and lignin) were significantly (*P*<0.05) higher in SEC than in MAT samples (Figure 1). This included genes encoding amylase and pullulanase (related to starch degradation), arabinofuranosidase and xylanase (related to hemicellulose degradation), cellobiase and exoglucanase (related to cellulose degradation), pectinase (related to pectin degradation), endochitinase and exochitinase (related to chitin degradation), and glyoxal oxidase and phenol oxidase (related to lignin degradation). These results indicated that the soil microbial metabolic capacity involved in carbon degradation were significantly different between the two different forest types.

**Figure 3:**
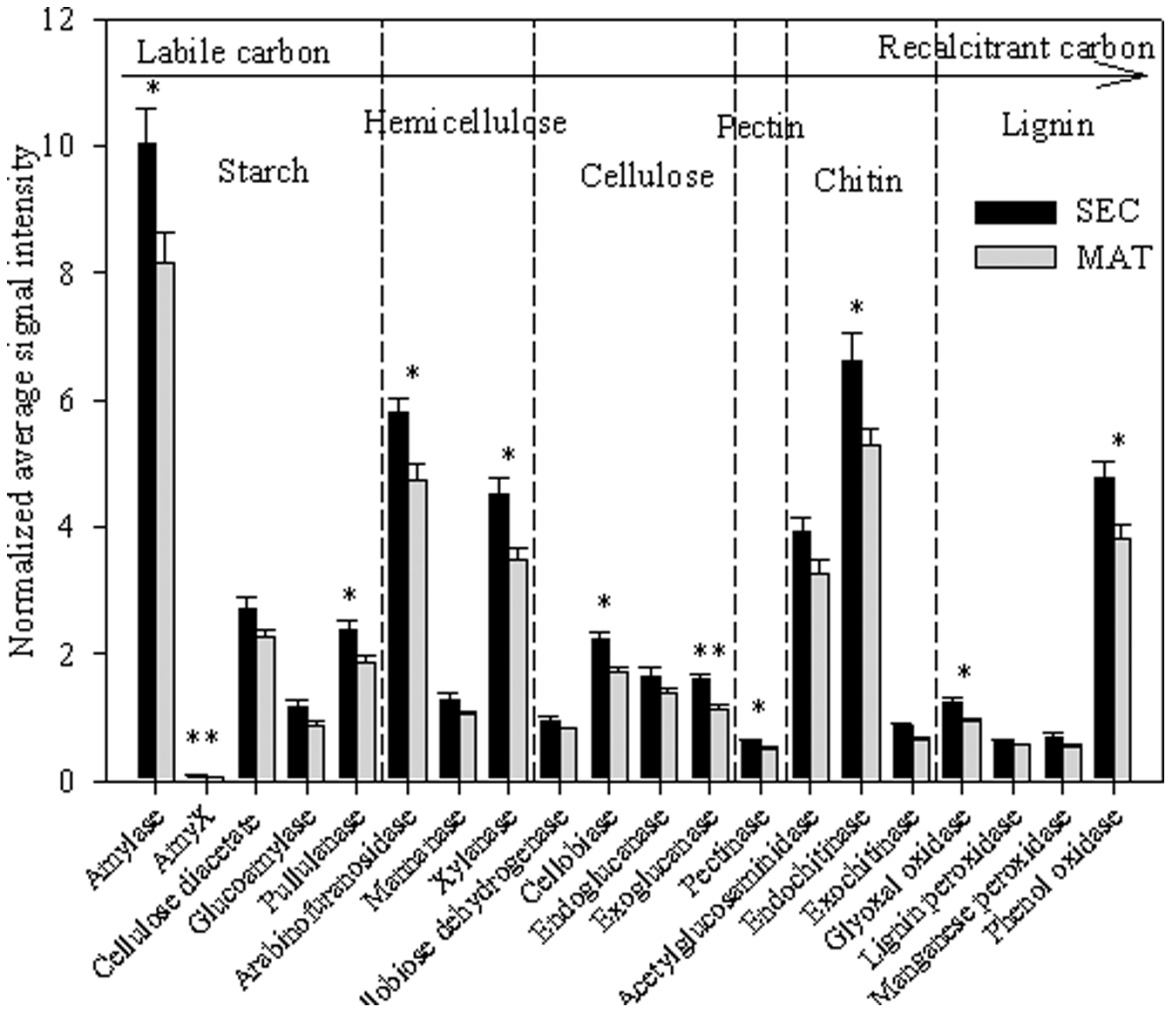
The normalized gene relative signal intensity of the detected key gene categories involved in carbon degradation. The signal intensities were the sum of detected individual gene for each functional gene, averaged among 4 soil samples. The complexity of carbon is presented in order from active to recalcitrant. All data are presented as mean ± standard error. Significant differences between two forest types are indicated above the bars. *, *P*<0.05, **, *P*<0.01.

Among the 1,085 genes involved in carbon fixation, the relative abundance of three important functional genes, ribulose-1,5-bisphosphate carboxylase/oxygenase (Rubisco), carbon monoxide dehydrogenase (CODH), and propionyl-coA (Pcc), was substantially different. A total of 306, 650 and 230 gene probes were detected for the Rubisco, Pcc, and CODH pathways, respectively. These genes had significantly higher signal intensity in SEC soils than in MAT soils (Figure S1). Geochip 4.0 also detected three enzymes involved in methane metabolism, namely the alpha-subunit of methyl coenzyme M reductase (mcrA), for methane production, and particulate methane monooxygenase (pmoA) and methane monooxygenase (mmoX) for methane consumption. Over 80% of the gene probes were associated with uncultured bacteria and archaea. The signal intensity of all the genes involved in methane metabolism was higher in SEC samples than in MAT samples (Figure 3). These results indicated that the microbial functional capacity related to carbon fixation and methane metabolism may be significantly affected by forest types. The relationships between soil microbial metabolic capacity related to carbon cycling and soil carbon components or enzyme activities were analyzed using the Mantel test (Table 3). The results showed that soil organic carbon and soil microbial biomass carbon were significantly linked (*P*<0.05) to the relative abundance of genes related to carbon cycling. Oxidizable organic carbon was significantly linked (*P*<0.05) to the relative abundance of genes involved in active organic carbon degradation (cellulase, hemicellulase, and starch). As expected, cellulase, glucanase, and amylase activities were significantly linked (*P*<0.05) to the relative abundance of genes encoding cellulase, endoglucanase and exoglucanase, and amylase, respectively.

**Table 3:** The relationships between relative abundances of microbial functional genes involved in carbon and nitrogen cycling and soil carbon or nitrogen components and enzyme activity by Mantel-test.

Soil carbon parameters	Microbial relative abundance	R value	P
Soil organic carbon	Carbon cycling genes	0.389	**0.039**
Oxidizable organic carbon	Oxidizable organic carbon degradation genes(Cellulose, Hemicellulose, Starch)	0.403	**0.046**
Water dissolved organic carbon	Carbon cycling genes	0.114	0.216
Soil microbial biomass carbon	Carbon cycling genes	0.500	**0.020**
Total soil nitrogen	Nitrogen cycling genes	0.382	**0.050**
NH_4_^+^-N	Ammonification genes	0.296	**0.089**
No_3_^–^N	Nitrification genes	0.302	**0.079**
Cellulase activity	Cellulase genes	0.342	**0.046**
Glucanase activity	Endo- and exoglucanase genes	0.351	**0.022**
Polyphenol oxidase activity	Polyphenol oxidase genes	0.046	0.626
Amylase activity	Amylase genes	0.358	**0.027**

### Microbial functional genes and metabolic potential related to nitrogen cycling

Among the gene probes detected in the forest soil samples, 4,238 were involved in nitrogen cycling, including 767 related to nitrogen fixation, 513 related to ammonification (including 250 gene probes related to assimilatory N reduction), 1,734 related to denitrification, 351 related to dissimilatory N reduction, and 621 related to nitrification. These key functional gene categories were associated with different phylogenetic groups, for example, almost all gene probes related to the ammonification were derived from cultured bacteria, including *Rhodococcus erythropolis*, *Pseudomonas entomophila*, and *Acinetobacter baumannii*. A total of 1,533 (88%) gene probes related to the denitrification were linked to unclassified bacteria, and 137 (22%) gene probes related to the nitrification were derived from archaea, which suggests that archaea maybe play an important role in nitrification process.

**Figure 4:**
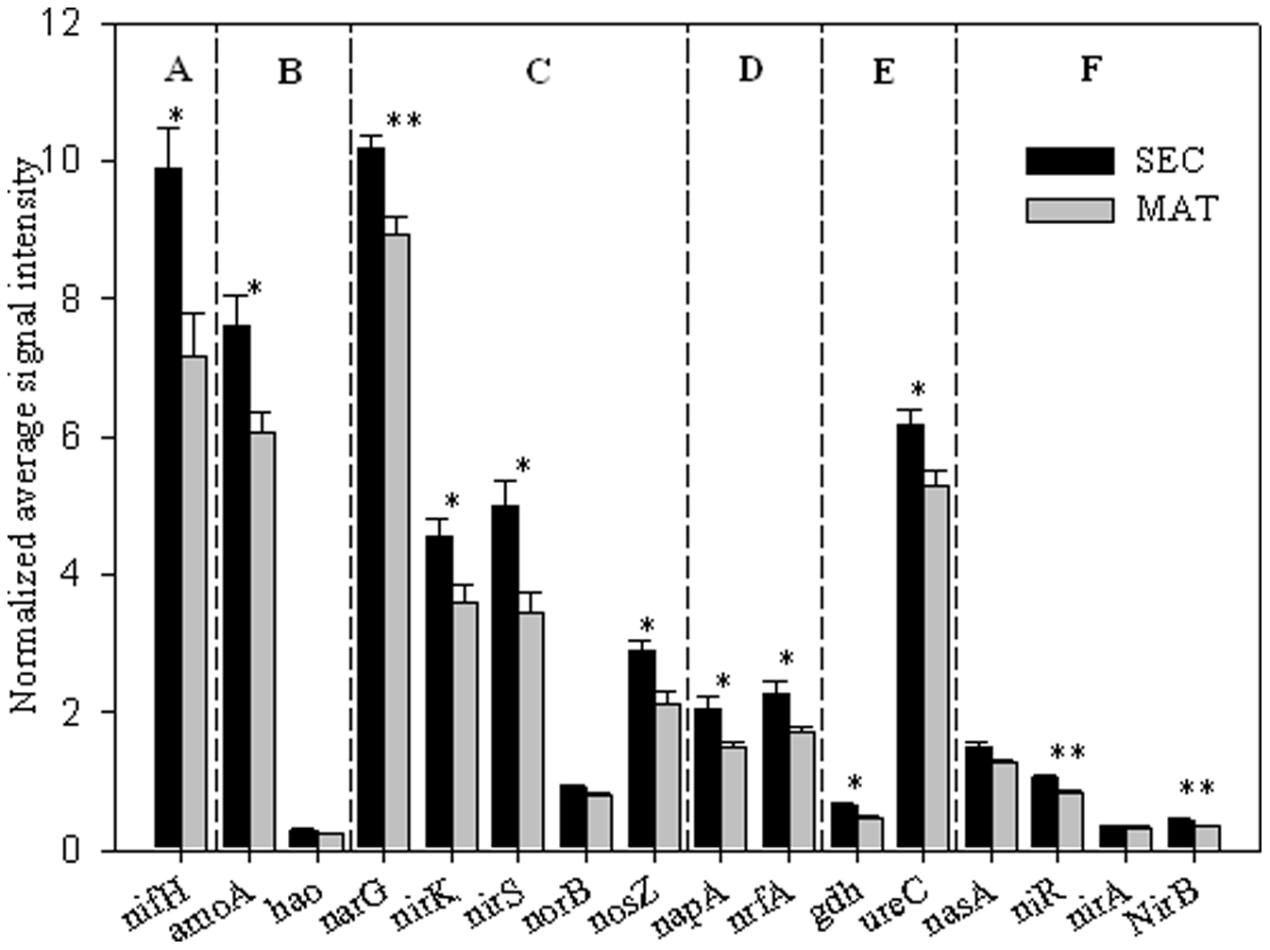
The normalized average signal intensity of detected key gene categories involved in the N cycling between SEC and MAT. The signal intensities were the sum of all detected individual gene for each gene category, and then averaged among 4 samples. (A). N_2_ fixation, including nifH encoding nitrogenase; (B). Nitrification, including amoA encoding ammonia monooxygenase, hao for hydroxylamine oxidoreductase; (C). Denitrification, including narG for nitrate reductase, nirS for nitrite reductase, norB for nitric oxide reductase, and nosZ for nitrate reductase; (D). Dissimilatory N reduction to ammonium, including napA for nitrate reductase, and nrfA for c-type cytochrome nitrite reductase; (E). Ammonification, including gdh for glutamate dehydrogenase and ureC encoding urease; (F). Assimilatory N reduction, including nasA encoding nitrate reductase, niR, nirA and nirB encoding dissimilatory nitrite reductase. All data are presented as the mean±SE (error bars). **, *P*<0.01, *, *P*<0.05.

The abundance of several genes involved in nitrogen cycling were significantly (*P*<0.05) higher in SEC samples than in MAT samples (Figure 4), including *nifH* (related to nitrogen fixation), *amoA* (related to nitrification), *narG*, *nirS*, *nirK*, and *nosZ*, (related to denitrification), *napA* and *nrfA* (related to dissimilatory N reduction to ammonium), *gdh* and *ureC* (related to ammonification), and *niR* and *nirB* (related to assimilatory N reduction). The relationships between microbial functional genes and soil nitrogen components were analyzed using the Mantel test (Table 4). The results showed that soil total nitrogen was significantly linked (*P*<0.05) to the relative signal intensity of nitrogen cycling genes. The NH_4_^+^-N and NO_3_-N concentrations were linked (*P*<0.1) to the signal intensity of genes involved in the ammonification and nitrification processes, respectively.

**Table 4:** Mantel test and partial Mantel test between 16S rRNA OTUs and Geochip data with environmental properties.

Environmental variable	16S OTUs		GeoChip 4.0	
	r value	P value	r value	P value
Whole variable	0.868	0.005	0.412	0.008
Soil organic carbon	0.770	0.003	0.392	0.013
Total nitrogen	0.832	0.003	0.402	0.019
pH	0.622	0.006	0.322	0.043
Temperature at the 10 cm depth	0.463	0.002	0.085	0.272
Shannon Index for trees and shrubs	0.779	0.006	0.337	0.028
Diameter at breast height of trees	0.532	0.008	0.125	0.158

The Mantel test was only performed for whole variable, which included all variables presented in this table.

### Linking microbial community structure and environmental factors

**Figure 5:**
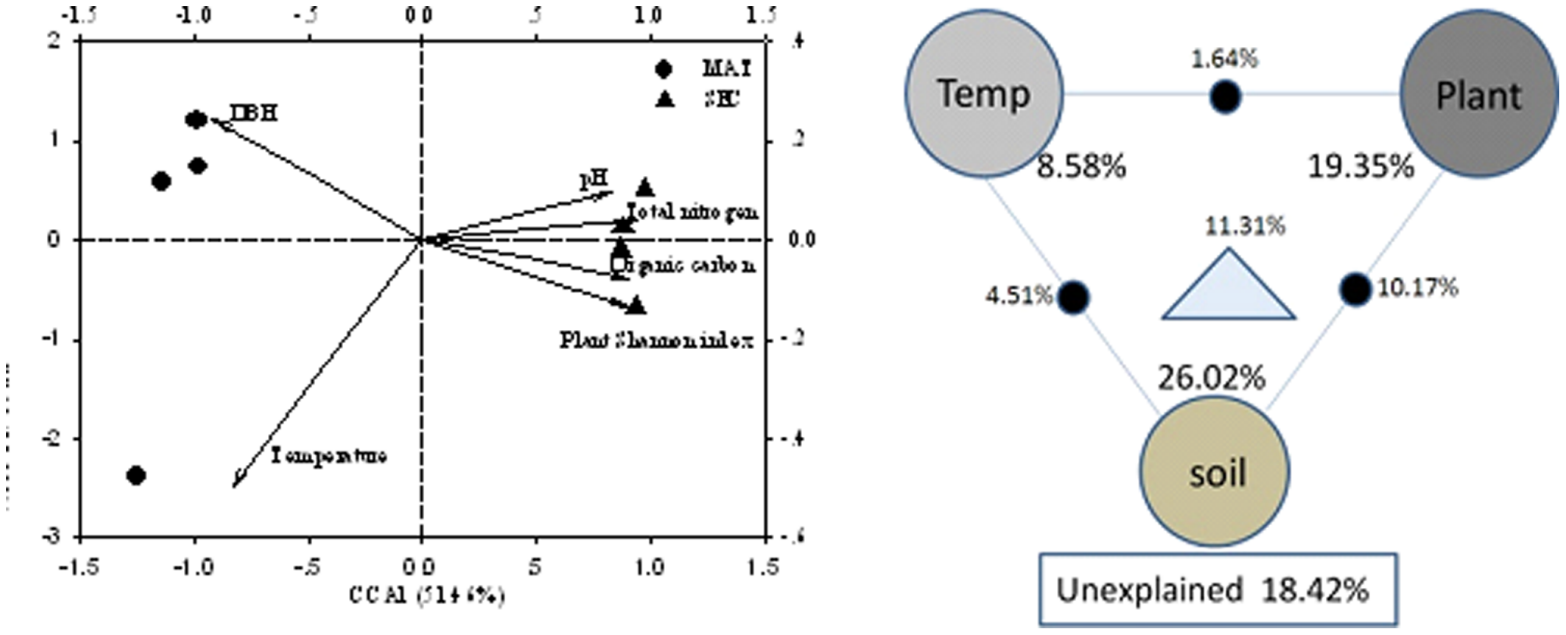
The Canonical correspondence analysis (CCA) and Variation partitioning analysis (VPA) between OTUs data and soil environmental variables. The left was CCA between microbial OTUs data and soil environmental factors, and the right was the VPA. DBH, diameter at breast height of tree. Soil, including soil organic carbon, total nitrogen and pH, soil temperature (Temp) and plant diversity (Plant, including the Shannon index of trees and shrubs, diameter at breast height of trees), and their relationships. Each diagram represents the biological variation partitioned into the relative effects of each factor or a combination of factors, in which geometric areas were proportional to the percentages of explained variation.

Mantel tests and CCA were performed to analyze the major environmental factors responsible for shaping the microbial community structure and functional diversity. The Mantel test showed that microbial community structure and functional gene diversity were significantly (*P*<0.05) linked to soil and plant factors (Table 4). CCA was used to identify the major environmental variables controlling the soil microbial community structure, resulting in a model significant at a confidence level of *P* = 0.048. The plant Shannon index and tree DBH appeared to be important environmental factors controlling the microbial community structure, as they had a long projection on Axis 1 (*P*<0.01), which represented the major variation among microbial communities (Figure 5A). Similarly, soil temperature and soil organic carbon were critical for shaping microbial communities.

Variance partitioning analysis was used to quantify the contributions of soil chemical factors (Soil), soil temperature (Temp), and plant diversity (Plant) to the variation in the microbial community. A total of 81.58% of the variation was significantly explained by these three environmental variables (Figure 5B). Soil, Temp, and Plant explained 26.02%, 8.58%, and 19.35% of variation, respectively, verifying that they were major factors in shaping the microbial community structure.

## Discussion

The development and increasing availably of high-throughput molecular techniques has helped improve our understanding of microbial composition, especially through the use of direct sequencing of metagenomic DNA. Fierer et al. indicated that all biomes were dominated by *Acidobacteria*, *Actinobacteria*, *Proteobacteria* and *Bacteroidetes*, and the bacterial community composition does not vary significantly across different biomes [1].Our results showed that microbial diversity at the phylum level differed between the two forest types, and *Acidobacteria*, *Protecobacteria*, and *Actinobacteria* were three of most abundant soil bacterial phyla. *Acidobacteria* is known to be dominant in other soil types, although they are often difficult to cultivate [8], [28]. The relative abundance of *Acidobacteria* was also shown to be higher in forest, desert, and prairie soils compared with agricultural soils [28]. Recent studies showed that *Acidobacteria* is in general oligotrophic, consistent with results showing that the proportion of *Acidobacteria* was significantly lower in nutrient-rich rhizosphere and agricultural soils than in bulk soil [4], [29]. Some studies also found that the relative abundance of *Acidobacteria* was negatively linked to the level of nitrogen input [30]. In this study, the relative abundance of *Acidobacteria* was high in the natural forest, which had low soil organic carbon and nitrogen contents.

Due to microbial highly diversity and natural ecosystem complexity, it is a great challenging to set up the link of soil microbial structure and composition to functional activity related to ecosystem functioning in microbial ecology. Many studies have described microbial community structure in a range of different environments, but have not identified critical groups related to detailed functional processes [4]–[8], [31]. Therefore, it remains unclear the relationship between microbial community structure and microbial functional gene diversity [32]–[34]. Functional redundancy is considerable debate in ecology as to how the relationship between the taxonomic diversity and observed functional diversity [35]. This study combines high-throughput sequencing to study the microbial community structure at the phylogenetic level and GeoChip to microbial potential metabolic activities at functional gene level. 16S rRNA gene-based high-throughput sequencing data provide information on microbial phylogenetic structure and composition, and GeoChip-based data provide quantitative information on various microbial functional groups [23]. These integrated technologies provide a reliable method for detection soil microbial structure, composition and potential functional activity [23]. In this study, a strong positive correlation were found between the soil microbial functional and phylogenetic diversity among sampling sites, which suggesting microbial phylogenetic and functional diversity aligned well and a low degree of functional redundancy were existed. Our result was consistent with a recent work on reconstructing the microbial diversity and function of pre-agricultural tallgrass prairie soils in the United States [36]. Furthermore, the higher phylogenetic diversity detected in SEC was consistent with the higher number of functional genes involved in the carbon and nitrogen cycles, unveiling the larger functional potential and capacity of the secondary forest soil.

Soil microbial metabolic potential and ecosystem function have received little attention owing to difficulties in methodology. As we all known, most of the biogeochemical processes were controlled and related to diverse and various functional genes, therefore, the conjunction between microbial community identified by using normal molecular methods (i.e. DGGE, T-RFLP) with soil enzyme activities or components is difficult. GeoChip-based data provides large-scale quantitative information on various biogeochemical functional groups, thus making it possible to link the functional structure of microbial communities with ecosystem processes [23]. Previous studies showed that change observed in the microbial community structure and functional groups has significant effects on soil enzyme activities or components related to carbon and nitrogen cycles. Yergeau et al. [37] and Reeve et al. [34] showed a significant correlation between cellulase enzyme activity and cellulose gene variants, detected using GeoChip, as well as significant correlations between dehydrogenase genes and enzyme activity, urease genes and soil ammonium, and sulfite reduction genes and soil sulfur level [34]. In this study, we also found the linked of soil microbial functional gene diversity to soil enzyme activities and components, such as the soil organic carbon and soil microbial biomass carbon were significantly linked (*P*<0.05) to the relative abundance of genes related to carbon cycling, oxidizable organic carbon was significantly linked (*P*<0.05) to the relative abundance of genes involved in active organic carbon degradation (cellulase, hemicellulase, and starch), respectively. Although the GeoChip data can't directly reflect the actual microbial populations and functional activities, it implied that the microbial populations carrying those genes could exist and have the functional capacity. Our GeoChip data showed that metabolic genes involved in the degradation of starch, cellulose, hemicellulose, chitin, lignin, and pectin, as well as those involved in nitrogen cycling, were significantly higher (*P*<0.05) in SEC than in MAT soils. Therefore, SEC soil had high microbial gene diversity and microbial metabolic potential. The use of GeoChip 4.0 may allow us to rapidly obtain community-wide information and observe differences functional capacity in different environments.

Only about 1% of the microorganisms in soil can be cultured, and researchers have increased our understanding of microbes in the natural environments by use of PCR amplification methods which reduce our dependence on the presence of culturable microbes. In our study, the majority of the genes related to carbon and nitrogen cycles were derived from the uncultured bacteria, suggesting that they are unique and may represent novel genes in the study area. For example, 88% gene probes related to the denitrification process were linked to unclassified bacteria. In previous studies, researchers received the same results, for example, Xie et al found 64.28% sequences were from uncultivated bacteria in the acid mine drainage [38], Zhang et al found the 320/372 genes were derived from the unidentified or uncultured organisms in the alpine meadows soils in the Qinghai-Tibetan plateau [39]. Therefore, a lot of the microbial functional gene resources were existed in natural environments.

Much recent work in soil microbial ecology has showed that soil microbial community structures are affected by specific environmental changes or disturbances [11], [25]. Soil properties and plant diversity are key factors in shaping the microbial community composition and structure [40], [41]. Organic carbon availability limited microbial communities in most soils [37], and addition of labile organic material rapidly alters microbial communities by selecting for populations that are most competitive in terms of growth rates and ability to absorb nutrients [41]. Thus, it was not considered to be the limiting factor for the microbial communities. Our data showed that plant diversity also significantly influenced the microbial community structure at the phylum level. Plant communities can influence the associated soil microbial communities through the types and amounts of organic carbon and nutrient inputs, and by altering the temperature and water content of the soil [42], [43]. Therefore, changes in plant diversity, composition, and production affect the composition and diversity of soil microbial communities [9]. However, our understanding of differences in soil microbial diversity depending on various vegetation types is still poor.

In summary, this work studied the microbial community structure and metabolic potential by combining high-throughput sequencing and GeoChip technologies, and showed the soil microbial community structure and functional metabolic potential had a positive correlation. Although the microbial populations and metabolic activities could not be characterized comprehensively, the detected functional gene groups may represent the *in situ* microbial metabolic potential at the functional cluster level to some degree. Further analysis of the functional activity with different approaches such as mRNA-based analysis is needed to understand the biogeochemical processes related to microbial community.

## Supporting Information

Figure S1 The normalized gene relative signal intensity of the detected key gene categories involved in carbon fixation and methane cycling. The signal intensities were the sum of detected individual gene for each functional gene, averaged among 4 soil samples. CODH: carbon monoxide dehydrogenase; Pcc: propionyl-coA; Rubisco: ribulose-1,5 -bisphosphate carboxylase/oxygenase; pmoA: methane monooxygenase; mcrA: methyl coenzyme M reductase. All data are presented as mean ± standard error. Significant differences between two forest types are indicated above the bars. *, *P*<0.05, **, *P*<0.01.
(DOC)

Table S1 16S rRNA diversity index. Summary of sequence numbers and OTUs, Shannon index and Simpson index for each sample at MAT and SEC based on sequencing data.
(DOC)

Table S2 The difference of soil microbial community composition and structure. Statistical analysis of differences in the microbial community composition and structure between SEC and MAT at the phylum level.
(DOC)

Table S3 The functional gene diversity index. Summary of numbers of detected gene probes, Shannon index and Simpson index for each sample at MAT and NAF based on GeoChip 4.0 data.
(DOC)

Table S4 The detected functional genes families. Summary of numbers of detected gene probes for different functional gene families at SEC and MAT based on GeoChip 4.0 data.
(DOC)
