# Supplementary material for: An Integrated Study to Analyze Soil Microbial Community Structure and Metabolic Potential in Two Forest Types
Source: PLoS One. 2014 Apr 17;9(4):e93773. doi: 10.1371/journal.pone.0093773 (PMC3990527; doi:10.1371/journal.pone.0093773)
Supplement: Table S1 — 16S rRNA diversity index. Summary of sequence numbers and OTUs, Shannon index and Simpson index for each sample at MAT and SEC based on sequencing data. (DOC) [file pone.0093773.s002.doc]

| Sample | No. of sequences | No. of OTUs (0.03) | Shannon Index | Simpson Index |
| --- | --- | --- | --- | --- |
| MAT1 | 16,919 | 3,695 | 8.06 | 2,673.28 |
| MAT2 | 15,521 | 3,672 | 8.07 | 2,735.86 |
| MAT3 | 16,659 | 3,732 | 8.07 | 2,689.72 |
| MAT4 | 16,567 | 3,393 | 7.97 | 2,436.64 |
| **Total MAT** | **16,416.50±307.66** | **3,623.00±77.66** | **8.05±0.02** | **2,633.88±67.07** |
| SEC1 | 16,552 | 4,362 | 8.26 | 3,357.31 |
| SEC2 | 16,970 | 4,396 | 8.26 | 3,328.81 |
| SEC3 | 16,060 | 4,550 | 8.30 | 3,521.86 |
| SEC4 | 16,002 | 4,571 | 8.31 | 3,539.60 |
| **Total NAF** | **16,396.00±227.66** | **4,469.75±53.03** | **8.28±0.01** | **3,436.90±54.61** |
