# Supplementary material for: An Integrated Study to Analyze Soil Microbial Community Structure and Metabolic Potential in Two Forest Types
Source: PLoS One. 2014 Apr 17;9(4):e93773. doi: 10.1371/journal.pone.0093773 (PMC3990527; doi:10.1371/journal.pone.0093773)
Supplement: Table S2 — The difference of soil microbial community composition and structure. Statistical analysis of differences in the microbial community composition and structure between SEC and MAT at the phylum level. (DOC) [file pone.0093773.s003.doc]

| phylum | MRPP | | adonis | |
| --- | --- | --- | --- | --- |
|  | δ | p | R^2^ | p |
| All phylotypes | 0.387 | 0.026 | 0.688 | 0.001 |
| *Acidobacteria* | 0.355 | 0.022 | 0.735 | 0.023 |
| *Actinobacteria* | 0.381 | 0.031 | 0.729 | 0.001 |
| *Armatimonadetes* | 0.560 | 0.034 | 0.589 | 0.001 |
| *Bacteroidetes* | 0.408 | 0.035 | 0.730 | 0.004 |
| BRC1 | 0.425 | 0.028 | 0.000 | 1.000 |
| *Chlamydiae* | 0.675 | 0.028 | 0.402 | 0.001 |
| *Chloroflexi* | 0.507 | 0.023 | 0.656 | 0.001 |
| *Crenarchaeota* | 0.457 | 0.030 | 0.650 | 0.001 |
| *Cyanobacteria* | 0.255 | 0.028 | 0.761 | 0.001 |
| *Firmicutes* | 0.440 | 0.039 | 0.567 | 0.014 |
| *Gemmatimonadetes* | 0.464 | 0.022 | 0.619 | 0.001 |
| *Nitrospirae* | 0.650 | 0.033 | 0.306 | 0.018 |
| *Planctomycetes* | 0.467 | 0.034 | 0.630 | 0.001 |
| *Proteobacteria* | 0.391 | 0.033 | 0.673 | 0.001 |
| *Verrucomicrobia* | 0.365 | 0.027 | 0.606 | 0.001 |
| WS3 | 0.670 | 0.029 | 0.000 | 1.000 |
| Total Unclassified | 0.414 | 0.045 | 0.664 | 0.001 |
