# Supplementary material for: An Integrated Study to Analyze Soil Microbial Community Structure and Metabolic Potential in Two Forest Types
Source: PLoS One. 2014 Apr 17;9(4):e93773. doi: 10.1371/journal.pone.0093773 (PMC3990527; doi:10.1371/journal.pone.0093773)
Supplement: Table S3 — The functional gene diversity index. Summary of numbers of detected gene probes, Shannon index and Simpson index for each sample at MAT and NAF based on GeoChip 4.0 data. (DOC) [file pone.0093773.s004.doc]

| Sample | No. of relative signal intensity | No. of detected genes | Shannon Index | Simpson Index |
| --- | --- | --- | --- | --- |
| MAT1 | 278,365.50 | 28858 | 10.27 | 28,691.14 |
| MAT2 | 319,940.40 | 33777 | 10.42 | 33,558.40 |
| MAT3 | 347,953.80 | 37001 | 10.52 | 36,760.13 |
| MAT4 | 342,520.60 | 36295 | 10.50 | 36,084.81 |
| **Average MAT** | **322,195.09±15,818.65** | **33982.75±1843.04** | **10.43±0.06** | **33,773.62±1,828.91** |
| SEC1 | 438,620.50 | 48,409 | 10.78 | 47,981.84 |
| SEC2 | 348,352.90 | 37,026 | 10.52 | 36,789.35 |
| SEC3 | 389,527.10 | 41,912 | 10.64 | 41,659.01 |
| SEC4 | 424,975.50 | 46,362 | 10.74 | 46,052.22 |
| **Average SEC** | **400,369.00±20,190.50** | **43,427.25±2,528.22** | **10.67±0.06** | **43,120.61±2,490.77** |
