# Supplementary material for: An Integrated Study to Analyze Soil Microbial Community Structure and Metabolic Potential in Two Forest Types
Source: PLoS One. 2014 Apr 17;9(4):e93773. doi: 10.1371/journal.pone.0093773 (PMC3990527; doi:10.1371/journal.pone.0093773)
Supplement: Table S4 — The detected functional genes families. Summary of numbers of detected gene probes for different functional gene families at SEC and MAT based on GeoChip 4.0 data. (DOC) [file pone.0093773.s005.doc]

| Functional process | No. of probes designed | Total^a^ | Average^b^ | | p (unpaired-test) |
| --- | --- | --- | --- | --- | --- |
|  |  |  | SEC | MAT |  |
| Antibiotic resistance | 3349 | 1747 | 1489.75±79.53 | 1190.00±60.16 | 0.026 |
| Bacterial phage | 1100 | 371 | 298.00±29.57 | 202.00±15.02 | 0.039 |
| Carbon degradation | 9033 | 5792 | 4885.25±329.47 | 3755.75.75±235.64 | 0.035 |
| Carbon fixation | 1762 | 1085 | 951.00±45.96 | 749.25±36.76 | 0.015 |
| Methane metabolism | 507 | 252 | 210.50±17.84 | 136.50±11.27 | 0.017 |
| Nitrogen cycling | 7552 | 4238 | 3714.25±212.27 | 2847.75±167.41 | 0.020 |
| Phosphorus utilization | 1378 | 727 | 639.25±40.55 | 499.00±31.31 | 0.037 |
| Stress | 21574 | 10511 | 8909.50±572.22 | 6843.25±377.64 | 0.028 |
| Sulfur cycling | 3254 | 2465 | 2062.50±166.97 | 1536.50±111.00 | 0.045 |
| Metal remediation | 9478 | 5639 | 4904.25±245.54 | 3927.25±201.09 | 0.023 |
| Contaminant degradation 17919 | | 11126 | 9778.25±453.81 | 7972.50±356.48 | 0.022 |
| Energy process | 862 | 492 | 426.50±26.02 | 344.25±14.10 | 0.042 |
| Virulence | 3732 | 1809 | 1531.00±88.63 | 1214.00±60.14 | 0.030 |
| Others ( gyrB, bchY) | 2492 | 1185 | 994.50±63.85 | 732.00±51.80 | 0.020 |
| **Total** | **83992** | **47525** | **43427.25±2528.22** | **33982.75±1843.04** | **0.026** |

^a^ Data represent total numbers of gene probes detected by GeoChip 4.0 across all 8 samples.

^b^ Data represent the mean value and standard error of detected gene probes using 4 samples in different forest types.
