# Supplementary material for: An Integrated Study to Analyze Soil Microbial Community Structure and Metabolic Potential in Two Forest Types
Source: PLoS One. 2014 Apr 17;9(4):e93773. doi: 10.1371/journal.pone.0093773 (PMC3990527; doi:10.1371/journal.pone.0093773)
Supplement: Supplementary file 5 [file pone.0093773.s001.doc]

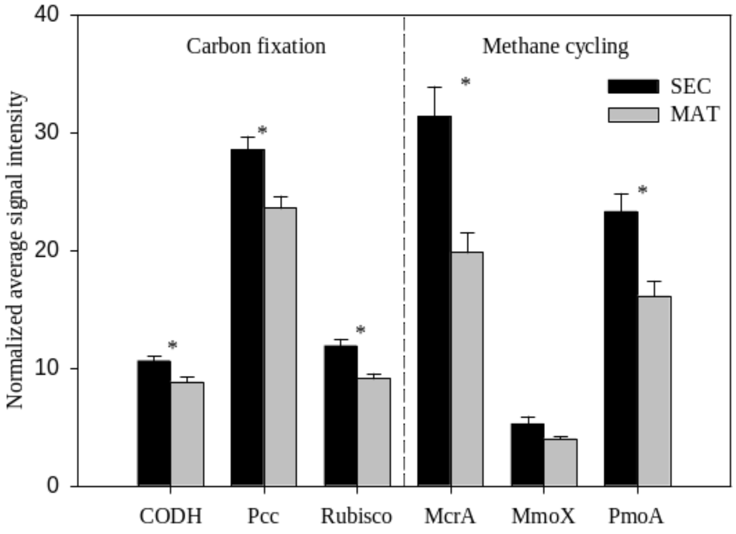

Carbon fixation
Methane cycling
SEC
MAT
Normalized average signal intensity
0
10
20
30
40
*
CODH
Pcc
Rubisco
McrA
MmoX
PmoA
